# On Relaxation of the Symphysis Pubis after Labor

**Published:** 1852-03

**Authors:** 


*On Relaxation of the Symphysis Pubis after Labor.* — It is not an uncommon occurrence after delivery, to find relaxation of the pubic ligaments; but from the uncertainty of the symptoms developed by it, it is liable to be mistaken for disease or displacement of the womb. M. Marten, (*Gazette Medicale*, Juliet,) hopes to throw some light upon the subject.

In all patients suffering from this affection, standing is peculiarly painful and difficult, so that the patient can barely walk a few paces even with the help of crutches. They also complain of more or less dysuria, with frequent micturition. If the hands are placed on the ilei while the patient attempts to walk, she will find that the hip of the leg which rests on the ground is pushed up, while the opposite, from contrast seems lower than natural.

In treating this accident, M. Marten makes use of a steel girdle, which entirely encircles the pubis. By means of the support thus afforded, he has known a patient walk with ease, who a few days previously could with difficulty stand upon her feet. — *Prov. Med. Jour.*

---

*Dr. John Haslam.*—An eccentric physician, lately deceased, (Dr. John Haslem,) one day declared, on his examination in a law court, that "For his part, he believed no one to be in perfectly sound mind, except the CREATOR." "And from what do you collect," said Mr. (now Lord) Brougham, wishing to make him utter some still more startling paradox, "that even HE is perfectly sane." "From my constant observation of the justice with which he wields his power," was the ready and serious reply.—*Lunacy and Lunatic Life.*

---

*Prurigo of the Genital and Anal Regions.* — Various remedies are from time to time recommended in the journals for this disagreeable and obstinate affection. The London Lancet, for December 15th, gives an application of M. Tournie's, as having been used with much success. "The affected spot is to be rubbed twice a day with calomel ointment, (one to two drachms of calomel to an ounce of axunge,) and, after each application, dredged with a powder, consisting of four parts of starch to one of powdered camphor." We have used a great variety of applications, for the relief of this unmanageable affection, and have derived most advantage from a cerate made of calomel (ʒi,) in Goulard's cerate (℥i.) — *Phil. Med. Examiner.*

